# Legius syndrome mutations in the Ras-regulator SPRED1 abolish its membrane localization and potentially cause neurodegeneration

**DOI:** 10.1016/j.jbc.2024.107969

**Published:** 2024-11-05

**Authors:** Yasuko Hirata, Hilde Brems, Seppe Van der Auweraer, Masaki Ohyagi, Mana Iizuka, Setsuko Mise-Omata, Minako Ito, Ludwine Messiaen, Seiya Mizuno, Satoru Takahashi, Eric Legius, Akihiko Yoshimura

**Affiliations:** 1 Department of Microbiology and Immunology, Keio University School of Medicine, Tokyo, Japan; 2 Department of Human Genetics, Catholic University of Leuven, Leuven, Belgium; 3 Division of Allergy and Immunology, Medical Institute of Bioregulation, Kyushu University, Fukuoka, Japan; 4 Medical Genomics Laboratory, Department of Genetics, University of Alabama at Birmingham, Birmingham, Alabama, USA; 5 Department of Anatomy and Embryology, Institute of Medicine, University of Tsukuba, Tsukuba, Japan; 6 Research Institute for Biomedical Sciences, Tokyo University of Science, Noda, Chiba, Japan

**Keywords:** SPRED1, Legius syndrome, membrane localization, palmitoylation, neurodegeneration, spermidine, autophagy

## Abstract

The SPRED family proteins act as negative regulators of the Ras-ERK pathway: the N-terminal EVH1 domain interacts with the Ras-GAP domain (GRD) of the NF1 protein, while the C-terminal Sprouty-related (SPR) domain promotes membrane localization of SPRED, thereby recruiting NF-1 to Ras. Loss-of-function mutations in the *hSPRED1* cause Legius syndrome in an autosomal dominant manner. In this study, we investigated the effects of missense mutations in the SPR domain identified in patients with Legius syndrome. Among the 18 mutations we examined, six (C368S, M369L, V408E, P415A, P415L, and P422R) have defects in the palmitoylation of the SPRED1 protein, losing plasma membrane localization and forming cytoplasmic granular aggregates. To evaluate the *in vivo* effects of SPR mutations, knock-in (KI) mice with P415A and P415V substitutions or M417Afs∗4, a C-terminal 28 amino acid deletion, were generated. All these KI mice exhibited cranial malformations, a characteristic feature of Legius syndrome. However, both P415A and P415V mutants formed granular aggregates, whereas M417Afs∗4 showed a diffuse cytoplasmic distribution, and *Spred1*^P415A^ and *Spred1*^P415V^ mice, but not *Spred1*^M417Afs∗4^ mice, developed cerebellar ataxia and Purkinje cell loss with age. These data suggest that in addition to loss of palmitoylation, the C-terminal region is required for the granular aggregate formation and Purkinje cell loss. The autophagy inducer spermidine rescued the ataxia phenotypes and Purkinje cell loss in *Spred1*^P415A^ mice. These results suggest that some, but not all, SPR mutations that lose lipid modification induce abnormal cytoplasmic aggregation, which could be a target for autophagic clearance, and potentially cause neurodegenerative diseases.

Germline loss-of-function mutations in the SPRED1 (Sprouty-related protein with an EVH1 domain-1) gene have been identified in patients meeting NIH clinical diagnostic criteria for neurofibromatosis type 1 (NF1) in whom NF1 mutations could not be identified; SPRED1 mutations represent at least 2% of pathogenic mutations in patients clinically diagnosed with NF1 (1, 2, 3). The phenotype of these patients, known as Legius syndrome (OMIM 611431), consists of multiple café-au-lait macules (CALMs), axillary freckling, macrocephaly, and sometimes mild neurocognitive deficits. Unlike NF1, Legius syndrome lacks neurofibromas, Lisch nodules, bony lesions, or optic nerve gliomas (3, 4). However, children with Legius syndrome may be at increased risk for leukemia (5). NF1, Legius syndrome, Noonan syndrome, Noonan syndrome with multiple lentigines (formerly known as LEOPARD syndrome), heart-facial-cutaneous syndrome, Costello syndrome, and SYNGAP1 syndrome collectively belong to a group referred to as RASopathies. These diseases are characterized by an increased Ras-ERK signaling pathway (6). Recently, recessive loss-of-function mutations in SPRED2 were found to cause a phenotype similar to Noonan syndrome (7).

SPRED family proteins, originally discovered as c-kit and c-fms binding proteins, have been shown to inhibit the Ras-ERK signaling pathway (8, 9). In mammals, four Sprouty homologs and three SPRED family members, SPRED1, SPRED2, and SPRED3, have been found (10). The C-terminal cysteine-rich (SPR) domain of SPREDs shows a similarity to that of Sproutys, and is palymitoylated (11). SPREDs are localized to the membrane fraction, in particular to the membrane raft through the SPR domain (12). The N-terminal enabled/vasodilator-stimulated protein homology 1 homology 1 (EVH1) domain of SPREDs binds to the NF1-GAP related domain (NF1-GRD), which reduces Ras-GTP levels (13, 14, 15). This elucidates the similarity of Legius syndrome to NF1 and the mechanism by which SPREDs inhibit the Ras-ERK pathway.

To date, more than 100 missense mutations in SPRED1 have been identified in Legius syndrome. Mutations in SPREDs are also discovered in various cancers including leukemia and mucosal melanoma (16). We and others have identified mutations in the EVH1 domain associated with Legius syndrome, as well as mutations in the GRD of NF1 patients that decrease the binding affinity between the GRD and the EVH1 domain (14, 15, 17). However, the significance of mutations in KBD (c-kit binding domain) and SPR has not been elucidated.

In this study, we identified several severe loss-of-function mutations within the SPR domain of SPRED1 in individuals with Legius syndrome. These SPR mutations interfere with its palmitoylation process, resulting in impaired localization to membrane rafts and reduced interaction with Ras. In addition, we observed the aggregation of these SPRED1 SPR mutations into punctate structures in the cytoplasm *in vitro*. To study the *in vivo* effects of a pathogenic SPR mutation, we generated P415A and P415V substitutions or M417Afs∗4, a frameshift mutation at M417 resulting in a C-terminal 28 amino acid deletion, were generated. P415A and P415V mutants formed cytoplasmic aggregates, whereas the C-terminal 28 amino acid deletion showed uniform cytoplasmic localization *in vitro*. Immunostaining of the wild-type (WT) mouse brain revealed Spred1 expression in Purkinje cells within the cerebellum. All three mutant mice exhibited phenotypes related to Legius syndrome, however, heterozygous P415A and P415V mutant mice developed cerebellar ataxia and Purkinje cell loss with age, whereas the C-terminal 28 amino acid deletion mutant did not. Based on our findings, we propose that SPRED1 not only functions as a typical Ras inhibitory molecule but also emerges as a candidate for autosomal dominant cerebellar ataxia.

## Results

### Reduced suppressive activity for the Ras-ERK pathway by SPR mutations

**Figure 1:**
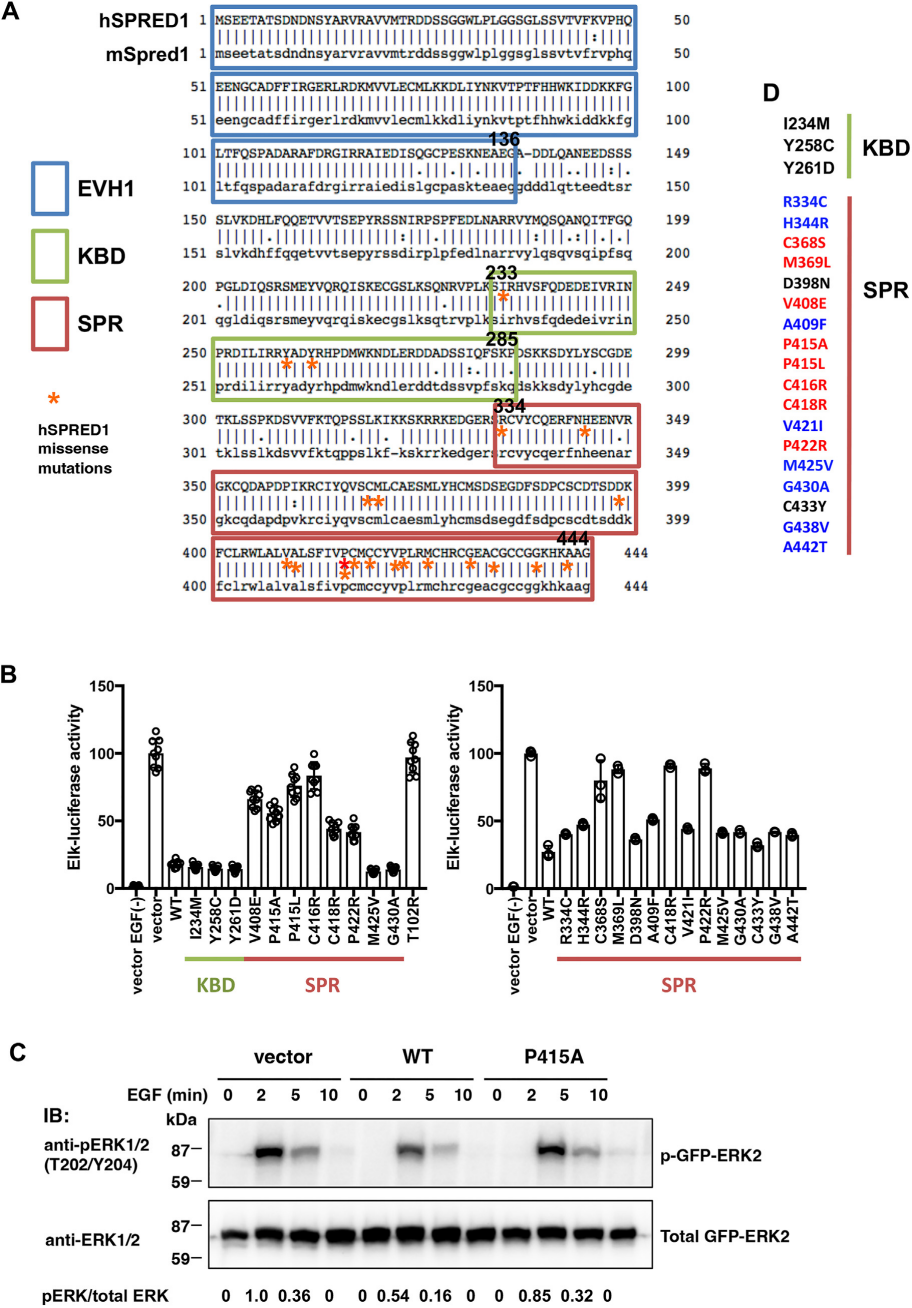
**Mutations of Legius syndrome examined in this study.***A*, comparison of the amino acid sequences of human SPRED1 and mouse Spred1. Domain assignment was based on the categorization of mSpred1 domains. The identity between human SPRED1 and mouse Spred1 is 93.0%, with a similarity of 94.6%, indicating a high degree of conservation. The asterisks indicate the positions of 21 mutations in hSPRED1 found in Legius syndrome that we investigated in this study, all of which were found to be conserved between humans and mice. *B*, suppression of EGF-induced Erk reporter activity by SPRED1 variants. As a loss-of-function control, the T102R mutation in the EVH1 domain was used. After 24 h transfection of 10 ng SPRED1 cDNAs and reporter genes, HEK293T cells were stimulated with 50 ng/ml EGF for 6 h, followed by the luciferase assay. The transfection efficiency was monitored by β-galactosidase activity. Elk reporter activity was normalized to an empty vector (vector) as 100%. The number of each group was six (*left panel*) and three (*right panel*) and the error bars represent the mean ± standard error of the mean (SD). The graphs show representative data from three independent experiments. *C*, detection of ERK phosphorylation. WT or P415A SPRED1 plasmids together with GFP-ERK2 cDNA were introduced into HEK293T cells, followed by EGF stimulation at a final concentration of 50 ng/ml for the indicated time periods. The ratio of the quantified density of the bands (pERK/total ERK) is shown at the bottom. Data are representative of three independent experiments. *D*, mutations in hSPRED1 that profoundly affect its ability to inhibit ERK upon EGF stimulation are highlighted in *red*, those that cause a slight decrease are highlighted in *blue*, and mutations that have no effect on ERK inhibition are highlighted in *black*.

We generated mutant SPRED1 cDNAs with three missense mutations present in the KBD and 18 mutations in the SPR domain discovered in patients with Legius syndrome and evaluated its Ras repression activity by ERK reporter assay (Fig. 1, *A* and *B*). The description of patient information is summarized in Fig. S1. The T102R mutant of the EVH1 domain lacks binding activity to NF1 completely lacks ERK inhibitory activity, and is used as a standard loss-of-function mutation. The results showed that the three mutations in KBD did not affect SPRED1 function at least by this assay. On the other hand, among the SPR mutations, C368S, M369L, V408E, P415A, P415L, C416R, C418R, and P422R showed a clear loss of inhibitory activity. Weak loss of inhibitory activity was also observed for R334C, H344R, A409F, V421I, G438V, and A442T, while D398N, M425V, G430A, and C433Y showed no apparent loss of function. Western blot analysis confirmed increased and prolonged ERK activation in response to EGF compared to WT SPRED1 (Fig. 1*C*). The effect of SPRED1 mutations on ERK suppression is summarized by color in Figure 1*D*.

**Figure 2:**
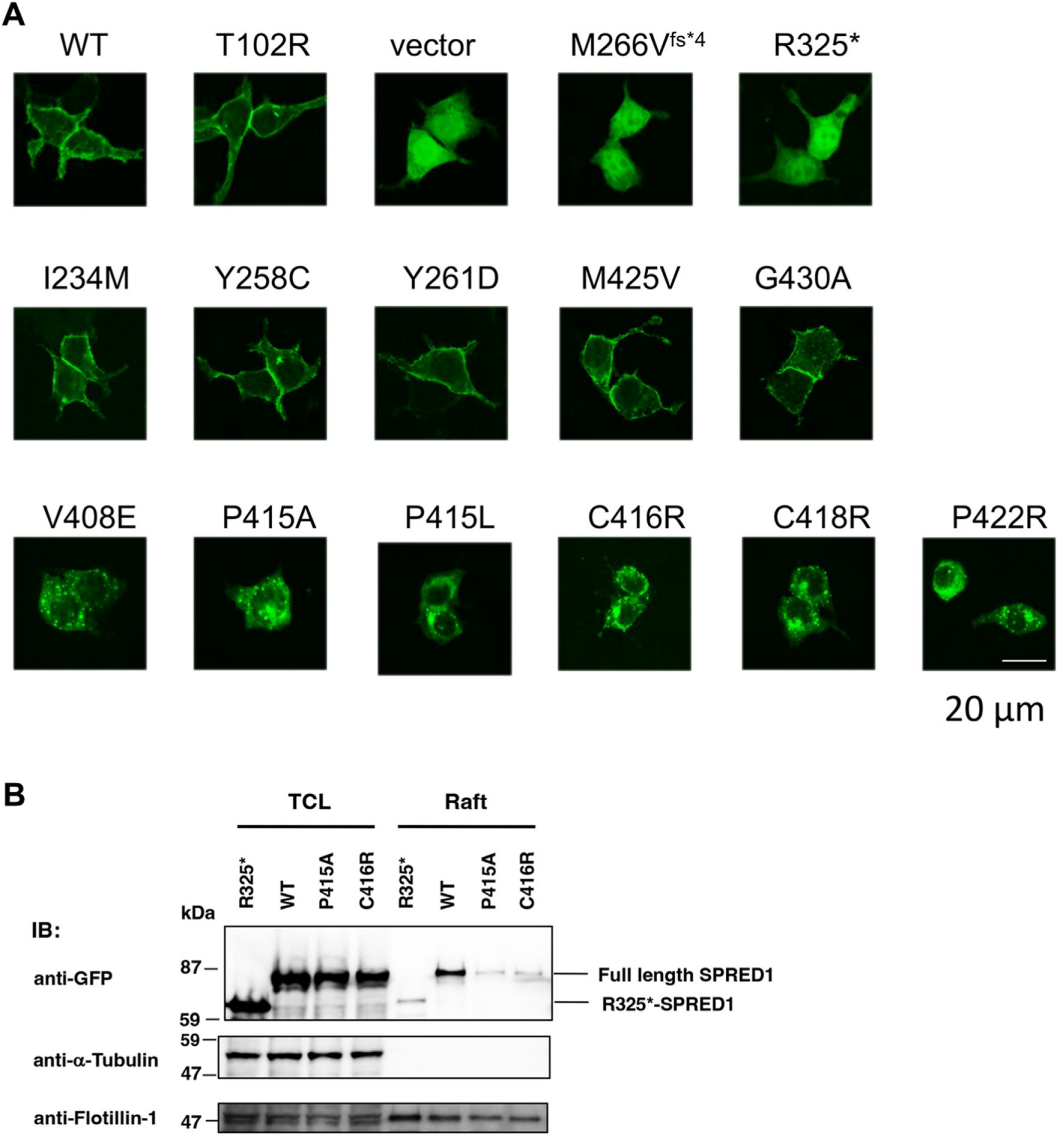
**Effect of SPRED1 mutations on membrane localization.***A*, fluorescence images of HEK293T cells expressing mutant SPRED1-EGFP. Representative images from at least three independent experiments are shown. Bar; 20 μm. *B*, membrane raft localization of WT, R325∗, P415A and C416R EGFP-tagged SPRED1. HEK293T cells were transfected with the indicated cDNA plasmids and the lipid raft fraction was obtained by sucrose density gradient centrifugation. Total cell lysates (TCL) before centrifugation and lipid raft fraction (Raft) were analyzed by Western blotting. α-Tubulin and flotillin-1 were used as non-raft and raft markers, respectively. The data are representative of three independent experiments.

Next, we examined the changes in intracellular localization due to SPR mutations by expressing SPRED1 mutants with an EGFP-tag in HEK293T cells (Fig. 2*A*). The WT and the T102R mutant localized to the cell membrane, whereas mutants of the SPR domain deletions, such as M266V^fs∗4^ (frameshift mutation at M266, resulting in C-terminal 179 aa deletion) and R325∗ (nonsense mutation at R325, resulting in C-terminal 120 aa deletion), were localized to the cytoplasm and nucleus. KBD substitutions (I234M, Y258C, Y261D) and those of SPR (M425V, G430A) which did not affect ERK activity, localized to the cell membrane. In contrast, mutants with decreased ERK inhibitory activity, such as V408E, P415A, P415L, C416R, C418R, and P422R, formed large granular aggregates in the cytoplasm. Previously, we have shown that SPRED1 localizes to membrane rafts *via* SPR (12). As shown in Figure 2*B*, the P415A and C416 mutants abolished raft localization.

### Severe loss-of-function SPR mutations have defects in S-palmitoylation

**Figure 3:**
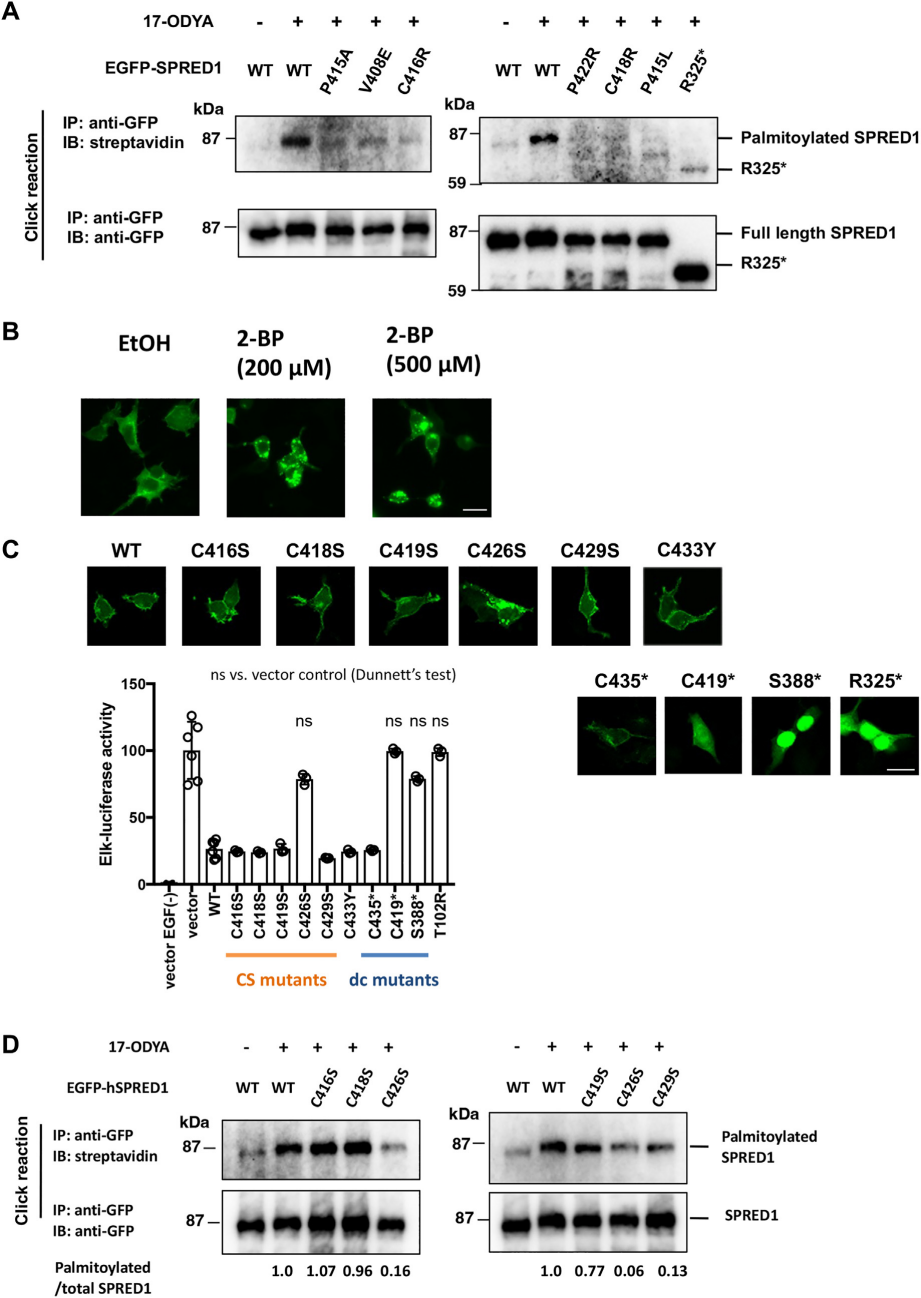
**Effect of SPRED1 mutations on palmitoylation.***A*, detection of palmitoylation of SPRED1 by metabolic labeling with an alkyne-containing palmitate analogue, 17-ODYA. HEK293T cells were transfected with plasmids carrying EGFP- hSPRED1 WT or SPR mutants were metabolically labeled with 17-ODYA for 14h. After EGFP- hSPRED1 protein was immunoprecipitated with anti-GFP agarose, followed by click chemistry was performed to introduce biotin into ODYA. Palmitoylated EGFP-SPRED1 was detected using streptavidin-HRP. The R325∗ mutant, which lacks the C-terminal SPR domain, was used as a negative control. *B*, inhibition of palmitoylation by 2-BP (2-bromohexadecanoic acid). WT EGFP-hSPRED1 was expressed in 293T cells on 4-well chamber slides and incubated with 2-BP (200μM or 500 μM) for 20 h after transduction. The cells were then fixed in 4% paraformaldehyde and the localization of WT EGFP-hSPRED1 was examined by fluorescence microscopy. Bar; 20 μm. *C and D*, effect of cysteine to serine substitution at the C-terminal region of the SPR domain on subcellular localization, ERK inhibitory activity, and palmitoylation. The indicated cysteine (C) to serine (S) substitution mutations and C-terminal deletion mutants, C435∗ (C-terminal 10 aa deletion), C419∗ (C-terminal 26 aa deletion), S388∗ (C-terminal 56 aa deletion) of EGFP-hSPRED1 were examined for their subcellular localization by fluorescence microscopy. Bar; 20 μm. ERK suppression activity was accessed by transfecting WT and mutant hSPRED1 plasmids together with Elk reporter plasmids. EGF-induced Elk reporter activity without SPRED1 vector was normalized to 100%. N = 3 for each transfection. In (*D*), palmitoylation of SPRED1 was detected by metabolic labeling with 17-ODYA as shown in (*A*). Representative data from at least two independent experiments are shown.

The SPR domain is known to localize to the cell membrane through palmitoylation (11, 18). To investigate whether SPR mutations affect palmitoylation, the alkyne-containing palmitic acid analog, 17-octadecynoic acid (17-ODYA), was added to the culture medium of SPRED1-expressing cells to metabolically label palmitoylated proteins in the cells (19). After SPRED1 protein was immunoprecipitated using anti-GFP antibodies, biotin-azide was subjected to click chemistry to introduce biotin labels to palmitoylated proteins. Using this method, biotin-labeled palmitoylated WT SPRED1 molecules were detected, whereas the R325∗ mutation (nonsense mutation at R325), which does not localize to the plasma membrane, showed no detectable palmitoylation. Furthermore, loss-of-function SPR mutations, including V408E, P415A, C416R, P422R, C418R, and P415L, showed almost no detectable palmitoylation in all cases (Fig. 3*A*). Importantly, WT SPRED1 forms intracellular aggregates upon treatment with 2-bromopalmitate (2-BP), an inhibitor of protein palmitoylation (Fig. 3*B*) (20). These data suggest that lack of palmitoylation is a primary cause of intracellular aggregates of SPRED1 protein.

Palmitoylation of proteins occurs on cysteine residues. Since the SPR domain is rich in cysteine residues, we sought to identify which cysteine residues are critical. The C-terminal 10 amino acid deletion mutant (C435∗) localized to the cell membrane, whereas the C-terminal 26 amino acid deletion (C419∗) did not, suggesting that the narrow C-terminal region of the SPR is palmitoylated. Therefore, we generated mutants in which cysteine residues in this region were replaced by serine (C416S, C418S, C419S, C426S, C429S). Among these mutants, C426S clearly lost its ERK inhibitory ability (Fig. 3*C*). Regarding intracellular localization, only the C426 mutant showed the formation of granular aggregates inside the cells. However, when measuring palmitoylation, C419S showed a slight decrease, and both C426S and C429S exhibited a significant, but not complete reduction in palmitoylation. This suggests that while there may be multiple palmitoylation sites, the palmitoylation of C426 is most critical for membrane localization. In addition, since the C419∗ lost plasma membrane localization but did not form cellular aggregation, the C-terminal 26 amino acids appear to be necessary for inducing cellular aggregates.

### Selective zDHHC proteins could be involved in palmitoylation of SPRED1

**Figure 4:**
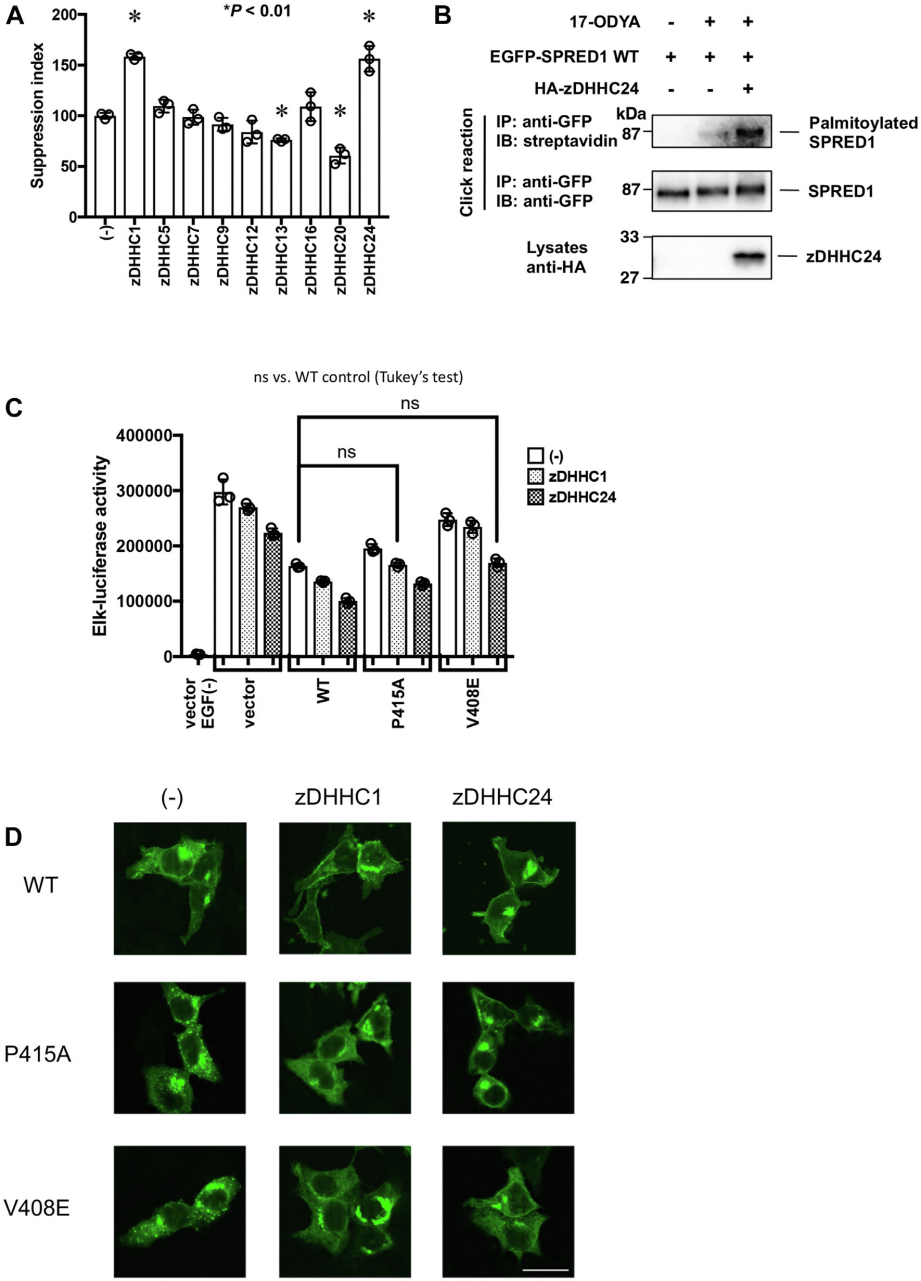
**Effect of zDHHC enzymes on SPRED1 localization and activity.***A*, HEK293T cells were transfected with 50 ng of each Elk-1 reporter, β-galactosidase gene, 10 ng zDHHC expression vectors, and 5 ng FLAG-tagged SPRED1 expression vectors. After 24 h, cells were stimulated with 50 ng/ml EGF for 6 h and then the activity of luciferase and β-galactosidase was analyzed. ∗∗*p* < 0.01 Dunnet’s test. The number of each group was three (N = 3), and the error bar represents mean ± SEM. *B*, effect of zDHHC24 overexpression on SPRED1 palmitoylation. Palmitoylation of SPRED1 was detected by metabolic labeling with 17-ODYA as shown in Figure 3*A*. *C*, effect of zDHHC1 or zDHHC24 on WT and SPR mutant SPRED1 activity. EGF-induced Elk reporter activity was measured in the presence or absence of SPRED1 and/or zDHHC plasmids. *D*, subcellular localization of WT and mutant EGFP-SPRED1 when co-expressed with zDHHC1 or zDHHC24. EGFP-hSPRED1 was examined for its subcellular localization by fluorescence microscopy. Bar; 20 μm.

In mammals, the palmitoylation (S-acylation) of intracellular proteins is catalyzed by 23 different zDHHC enzyme isoforms. According to recent reports by Butler *et al.*, the SPR domains of Sprouty2 and SPRED3 proteins interact with zDHHC17 to promote S-acylation (18). Therefore, we cloned several of the zDHHC enzymes and investigated their effect on the ERK inhibitory activity of SPRED1 by co-expression in HEK293T cells (Fig. 4*A*). Among the zDHHC genes we cloned, zDHHC1 and zDHHC24 positively influenced the function of WT SPRED1, *i.e.*, increased the ERK inhibitory activity of SPRED1. Other zDHHC enzymes either had no effect on SPRED1 activity on ERK or, in some cases, decreased its inhibitory activity. In addition, as shown in Figure 4*B*, co-expression of zDHHC24 strongly enhanced the palmitoylation of WT-SPRED1. We also confirmed the association of human SPRED1 with zDHHC24 in HEK293T cells through the SPR domain (Fig. S2*A*). The C-terminal 56 amino acid deletion (S388∗), but not the C-terminal 26 amino acid deletion (C419∗), of murine Spred1 disrupted binding to zDHHC24, suggesting that zDHHC24 binds to the region corresponding to S388 to 418C (Fig. S2, *B* and *C*).

Then we examined the effects of zDHHC1 and zDHHC24 on SPR mutants that aggregate intracellularly. As shown in Figure 4*C*, zDHHC1 had a weak effect, whereas zDHHC24 significantly improved the ERK inhibitory ability of the P415A and V408E mutant variants. Furthermore, examination of intracellular localization revealed that forced expression of zDHHC1 and zDHHC24 resulted in the partial localization of P415A and V408E mutant variants to the cell membrane (Fig. 4*D*). Based on these results, it was proposed that certain zDHHC molecules, including zDHHC1 and zDHHC24, are potential palmitoyl acyltransferases for SPRED1.

### P415 heterogeneous mutations induce loss of Purkinje cells and ataxia in mice

**Figure 5:**
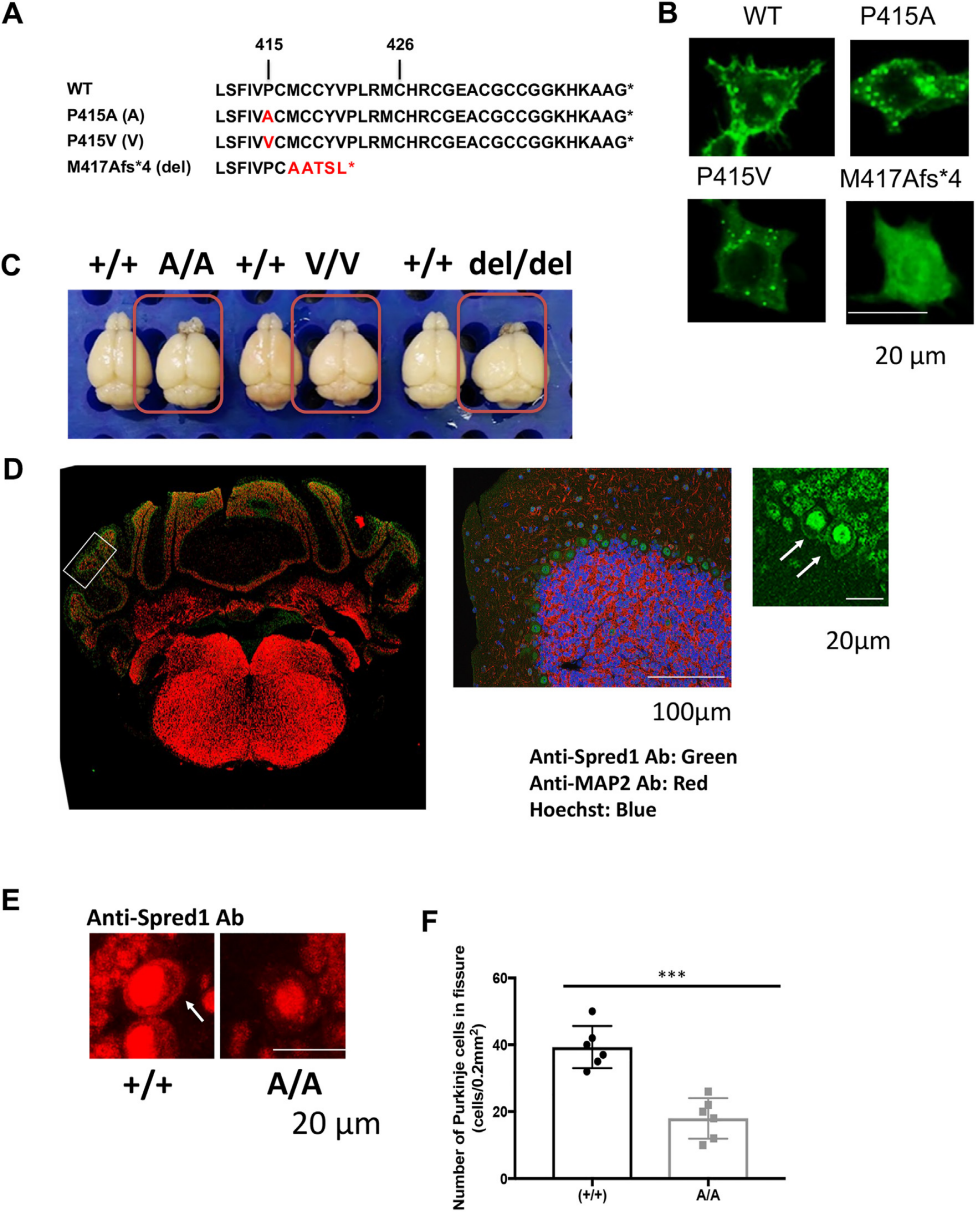
**Generation of Spred1-P415 knock-in (KI) mice.***A*, the P415A substitution was designed by homologous recombination using oligo-DNA and CRISPR/Cas9. As by-products, a P415V substitution and a 4 bp deletion (M417Afs∗4) resulting in a frameshift that lacked C-terminal 28 amino acids were also obtained. *B*, subcellular localization of EGFP-labeled P415A, P415V, and M417Afs∗4 mutants of murine Spred1 expressed in HEK293 cells. Bar; 20 μm. *C*, brain deformities of 14-week-old homozygous *Spred1*^P415A/P415A^ (A/A), *Spred1*^P415V/P415V^ (V/V), and *Spred1*^M417Afs∗4/M417Afs∗4^ (del/del) mice. *D*, immunostaining of the brain from 8-week-old BL6 male mice with SPRED1 antibody (*green*) and anti-MAP2 antibody (*red*). The arrows indicate membrane localization of WT SPRED1 protein. Bars; 100 μm and 20 μm, respectively. *E*, enlarged view of anti-Spred1 antibody staining (*red*) of WT and A/A mutant the Purkinje cells of the cerebellum. *Arrows* indicate membrane localization of Spred1 protein. *F*, the number of cerebellar Purkinje cells in WT and A/A mutant mice (19-month-old male mice; N = 6). Unpaired *t* test ∗∗∗*p* < 0.0001.

The granular intracellular protein aggregates observed in the SPR loss-of-function mutations are similar to neurotoxic aggregates associated with neurodegenerative diseases such as Alzheimer's disease (AD), Parkinson's disease, and Huntington's disease (HD). SPRED1 is highly expressed in the brain, and the enzyme zDHHC17 (HIP14), which is involved in the palmitoylation of mutant huntingtin (HTT), the causative gene for HD, has been reported to also be involved in the palmitoylation of Sprouty2 and SPRED3 (18). Therefore, to investigate whether SPR loss-of-function mutations could lead to neurodegenerative diseases, we generated Spred1-P415 knock-in (KI) mice using homologous recombination with oligo-DNA and CRISPR/Cas9 (Fig. S3). As by-products, a P415V substitution and a 4 bp deletion resulting in a frameshift (M417Afs∗4) were also obtained (Fig. 5*A*). M417Afs∗4 mutation resulted in a C-terminal 28 amino acid deletion. Similar to previous results shown in Figure 2, both P415A and P415V mutants formed granular aggregates in HEK293T cells, while M417Afs∗4 showed a diffuse cytoplasmic distribution (Fig. 5*B*). These three lines were backcrossed at least 6 times with WT C57BL6 mice.

The homozygous *Spred1*^P415A/P415A^ (A/A), *Spred1*^P415V/P415V^ (V/V), and *Spred1*^M417Afs∗4/M417Afs∗4^ (del/del) mice exhibited a pronounced phenotype with skull deformities, similar to Spred1 complete knockout (Spred1^−/−^) mice (Fig. 5*C*) (21). Immunostaining of the brain with SPRED1 antibody revealed plasma membrane localization of WT Spred1 in the Purkinje cells of the cerebellum (Fig. 5*D*). Dense nuclear staining was also observed with the antibody, but since knockout mice also showed similar staining, the nuclear staining is likely to be a non-specific one. As expected, Spred1^P415A/P415A^ protein was not detected in the plasma membrane of Purkinje cells (Fig. 5*E*).

**Figure 6:**
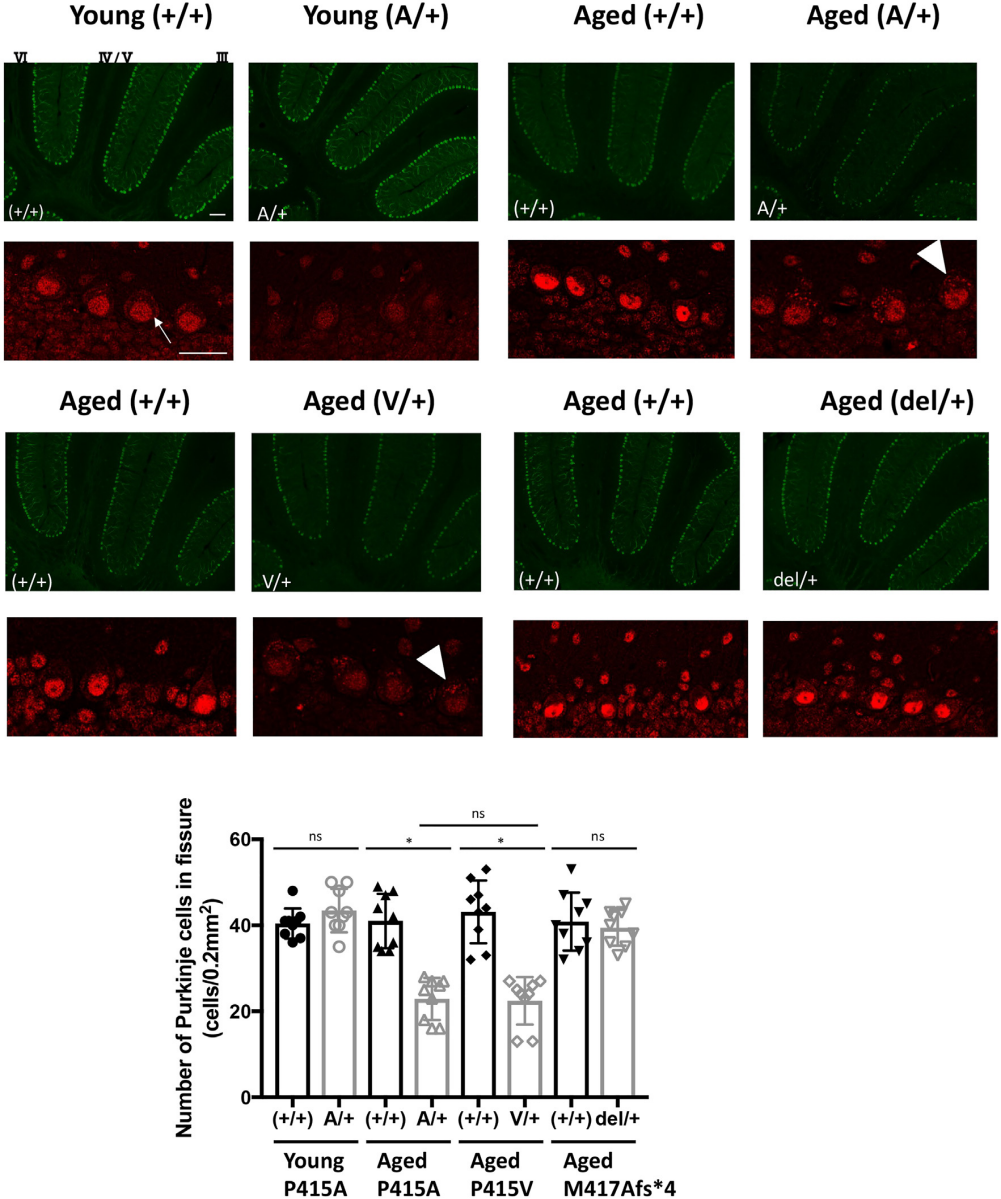
**Cerebellar Purkinje cells in WT and heterozygous *Spred1***^**P415A/+**^**(A/+), *Spred1***^**P415V/+**^**(V/+), and *Spred1***^**M417Afs∗4/+**^**(del/+) mice.** (*Upper panels*) Purkinje cells in the cerebellum were stained with anti-calbindin antibody (*green*) and anti-Spred1 antibody (*red*) in 5-month-old (young) and 19-month-old (aged) KI heterozygous mice and their littermates (+/+). The number of cerebellar vermis lobules is expressed as a Roman number. Bars; 100 μm (*green*) and 20 μm (*red*), respectively. The arrow indicates membrane localization of WT SPRED1 protein and arrow heads indicate intracellular aggregates of mutant SPRED1. (*Bottom panel*) Quantitative counting of cerebellar Purkinje cells. Three sections from three mice per group were used. Data are expressed as the mean ± SEM ∗*p* < 0.05 (Tukey's test).

A comprehensive analysis of heterozygous mice was then performed. Heterozygous *Spred1*^P415A/+^ (A/+), *Spred1*^P415V/+^ (V/+), and *Spred1*^M417Afs∗4/+^ (del/+) mice exhibited a mild but significant phenotype characterized by pronounced cranial deformities (Fig. S4*A*). However, no differences in body weight were observed between WT and heterozygous mice (Fig. S4*B*). We observed a reduction in the number of Purkinje cells in the cerebellum of A/+ and V/+ but not del/+ heterozygous mice, especially after 19 months of age. Interestingly, A/+ and V/+, mice at 5 months of age did not show Purkinje cell dropout (Fig. 6 and data for V/+ not shown). Similar Purkinje cell dropout was observed in A/A homozygous mice at 19 months of age (Fig. S4*C*).

**Figure 7:**
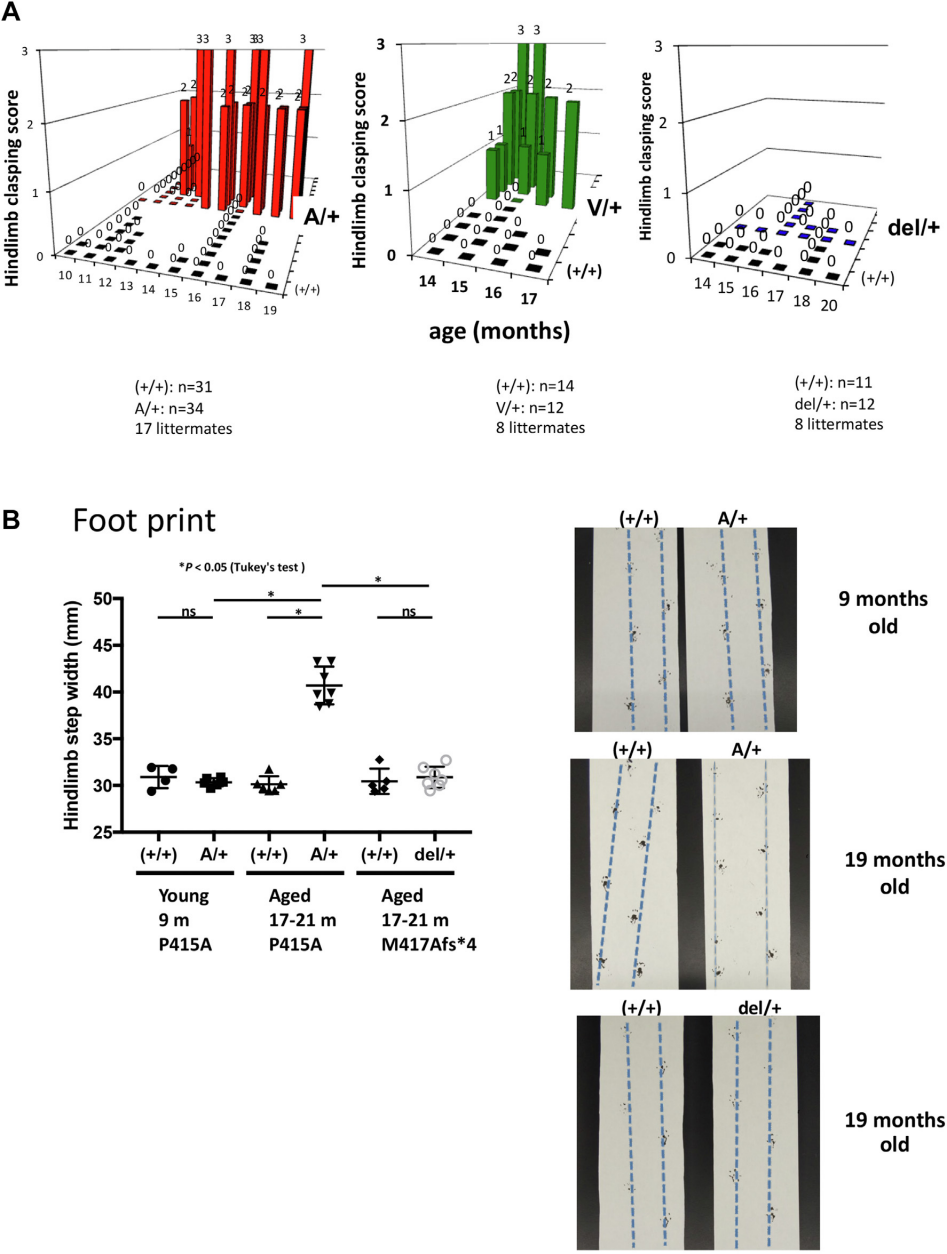
**Cerebellar ataxia symptoms of aged *Spred1***^**P415A/+**^**(A/+), *Spred1***^**P415V/+**^**(V/+) mice, and *Spred1***^**M417Afs∗4/+**^**(del/+) mice.***A*, the hind-limb clasping test. The number of mice tested is shown. *B*, footprint test. For the footprint assay, the step width of the hindlimb (mm) was measured (*left panels*). N = 4 to 7. Representative footprint patterns of WT (+/+) and heterozygous (A/+) or (del/+) mice are shown (*right*). Data are expressed as the mean ± SEM ∗*p* < 0.05 (Tukey's test).

Purkinje cell dropout is often associated with ataxia. Heterozygous mice did not show clear motor impairments at a young age, but at 14 months and older, A/+, V/+ heterozygous mice showed symptoms of cerebellar ataxia in the hind limb clasping test (Figs. 7*A* and S5) as well as in the walking test (Fig. 7*B*). In contrast, del/+ heterozygous mice did not show such symptoms.

### Induction of autophagy rescues ataxia phenotypes caused by P415A mutation

It has been known that intracellular aggregates in neural cells are degraded and removed by autophagy (22). Indeed, SPR mutant SPRED1 aggregates partly co-localized with p62/SQSTM1, an inducer of selective autophagy (Fig. S6) (23), suggesting that SPRED1 intracellular aggregates may be cleared by autophagy. Spermidine, an endogenous polyamine often used as a supplement, is known to activate autophagy (24, 25), and its effects on neurodegenerative diseases have been investigated (26, 27). For instance, oral administration of spermidine to APPPS1 mice, a model of AD, resulted in a reduction of soluble amyloid β (Aβ)-associated neurotoxicity and decreased AD-related neuroinflammation (28).

**Figure 8:**
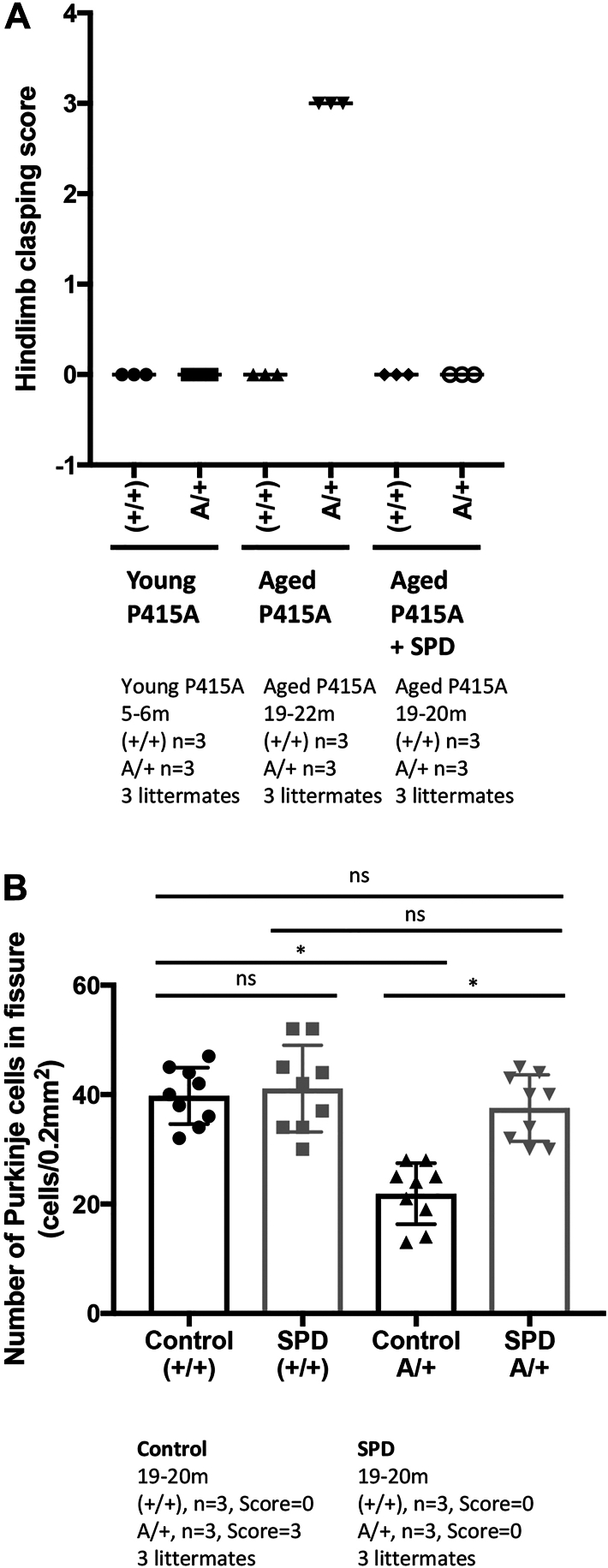
**Effect of spermidine (SPD) treatment on ataxia symptoms (*A*) and cerebellar Purkinje cell number (*B*) in WT (+/+) and heterozygous *Spred1***^**P415A/+**^**(A/+) mice.***A*, the hind-limb clasping test of young (5–6 months old), aged (19–20 months old) (+/+) or (A/+) mice. SPD (3 mM) in water was administered *ad libitum*. Control animals received normal autoclaved drinking water. Eight to nine 11- to 12-month-old mice were treated with SPD daily for 3 months, then every 2 weeks for a total of 8 months (*A*) The hind-limb clasping test was performed. *B*, Purkinje cells in the cerebellum were stained with an anti-calbindin antibody and quantified as shown in Figure 6. *p* < 0.05 (Tukey's test).

Therefore, an experiment was conducted in which 1-year-old A/+ mice received spermidine in their drinking water for 8 months. The results showed that spermidine administration did not alter the brain morphology of A/+ mice, but a significant improvement was observed in the hindlimb clasping test (Fig. 8*A*). Furthermore, spermidine administration suppressed Purkinje cell dropout (Figs. 8*B* and S7). These results strongly suggest that mutations in SPRED1 SPR domain lead to the formation of intracellular aggregates in neuronal cells, which overwhelm the autophagic clearance mechanism, resulting in Purkinje cell death and exhibiting cerebellar ataxia symptoms.

## Discussion

This study demonstrated that the SPR domain of SPRED1 undergoes S-acylation, presumably S- palmitoylation and that several missense mutations in Legius syndrome impair the membrane localization of SPRED1 due to defective palmitoylation, leading to reduced Ras inactivation. S-acylation is likely to occur at multiple cysteine residues within the SPR domain, but we found that S-palmitoylation of C426 appears to be critical for membrane localization. S-acylation of Sprouty2 by zDHHC17 has been reported to be dependent on C265 and C268 (29). This study showed that C265 is the most preferred S-acylation site, while C268 is also S-acylated, especially when C265 is mutated. Similarly, we found that both C426 and C429 appear to be palmitoylated; however, C426 is critical for membrane localization. There is no amino acid similarity between the region around C265 of Sprouty2 and that of C426 of SPRED1. Mutations in the SPR domain found in Legius syndrome are scattered and may affect binding to the S-acylation enzyme zDHHC and its substrate compatibility. While zDHHC17 has been shown to interact with the SPR domain of SPRED3 (18, 19), the detailed binding mode at the amino acid level remains unclear. Although a selectivity of zDHHC for SPRED1 has been suggested by various zDHHC overexpression experiments, knockdown experiments are needed to identify the physiologically relevant S-acylation enzyme.

Many proteins involved in neurodegenerative diseases are palmitoylated, and palmitoyl acyl transferase enzymes have also been implicated in a number of diseases (30). In this study, we also identified mutations that cause SPRED1 protein aggregation in the absence of lipid modification. Interestingly, simple deletion of the C-terminus of SPRED1, such as M417Afs∗4 (C-terminal 28 aa deletion) and C419∗ (C-terminal 26 aa deletion), resulted in loss of membrane localization but not cytoplasmic aggregation. Thus, C-terminal amino acids appear to be essential for aggregation, and the specific structural elements contributing to aggregation require further investigation.

In this study, we generated KI mice, focusing on the P415 mutation as a representative example, to investigate the significance of mutation-induced SPRED1 protein aggregation. Homo- and heterozygous P415A and P415V KI mice exhibited cranial deformities similar to SPRED1^−/−^ mice, highlighting the dysfunction of Ras inhibition. These mice also exhibited age-related cerebellar ataxia-like symptoms associated with Purkinje cell loss. In contrast, the M417Afs∗4 mutation, which results in a loss of 28 amino acids from the C-terminus, showed a phenotype of cranial malformations but not cerebellar ataxia. This is consistent with our hypothesis that mutations that induce SPRED1 protein aggregation lead to a neurodegenerative disorder. Although cerebellar ataxia symptoms are clearly observed, further investigations are needed to explore other neurodegenerative disorders such as dementia. A recent Genome-wide association study suggested that SPRED2 is linked to Alzheimer's disease (31). Unfortunately, there are no reported cases of patients with Legius syndrome and SPR mutations showing cerebellar ataxia or neurodegenerative symptoms. This may be due to the need for aging factors for clear symptoms, or perhaps the possibility of neurodegenerative symptoms was not previously considered. Our KI mice could provide a useful tool to investigate the role of SPRED1 in neurodegenerative disease. Detailed studies in human patients are warranted in the future.

In this study, we also examined three mutations identified in KBD; however, our assay did not provide insight into the significance of these mutations. Y228 and Y231 of Spred2 in the KBD have been identified as major tyrosine phosphorylation sites (32). Y258 and Y261 of SPRED1 correspond to these sites, suggesting that these two tyrosine residues are likely to be phosphorylated, thus playing a critical role in SPRED1 function. To investigate the role of KBD, we replaced the EGF receptor with c-kit in the ERK assay, since KBD is a domain that binds to c-kit. Surprisingly, the forced expression of KBD mutations suppressed SCF-induced ERK activation more effectively than WT SPRED1 (unpublished data). Phosphorylated Y228 and/or Y231 of Spred2 have been identified as sites for interaction with Cbl, leading to Spred2 protein degradation (32). This suggests that these KBD mutations may also increase SPRED1 expression levels. Furthermore, it has been reported that the extended KBD in SPRED1 and SPRED2 associates with SHP2, suggesting a potential involvement of this region in the regulation of the Ras-ERK pathway (33). It is noteworthy that gain-of-function mutations of SHP2 have been found in Noonan and LEOPARD syndromes (34). However, the significance of KBD mutations, especially in the *in vivo* context, requires careful consideration.

## Experimental procedures

### Patient SPRED1 cDNA

*SPRED1* mutation analysis was performed in the Department of Human Genetics, Catholic University of Leuven, Belgium, and in the UAB Medical Genomics Laboratory, University of Alabama at Birmingham, in individuals with a Legius syndrome phenotype (2). Some samples were sent by clinical geneticists from other centers. Individuals showed a phenotype compatible with Legius syndrome; specifically, the presence of CALM and/or freckling and the absence of neurofibromas. All patients in this study carry missense *SPRED1* variants with no *NF1* mutations and are listed in the SPRED1 LOVD database ((LOVD https://grenada.lumc.nl/LOVD2/mendelian_genes/home.php?select_db = SPRED1).

Mutation numbering was based on the cDNA sequence with +1 corresponding to the A of the ATG translation initiation codon in the reference sequence (GenBank accession code: NM_152594.2). For protein numbering, the initiation codon was codon 1 (NP_689807.1).

Human *SPRED1* cDNAs were cloned in pcDNA3 with a six-repeated Myc-tag or FLAG-tag in pCMV2 or EGFP-tag at the N-terminus using pEGFP-(C2) vectors (12, 14).

### Ethics information

This study was approved by the Institutional Review Board of Keio University School of Medicine (Tokyo, Japan; approval number 20120039), and the local institutional review board (IRB) of the Catholic University of Leuven and the University of Alabama at Birmingham. All experiments involving mice were approved by the Institutional Animal Care and Use Committee (IACUC; approval number, A2022–324) of Keio University and performed according to the IACUC guidelines.

The human studies reported in this manuscript abide by the Declaration of Helsinki principles.

### Other plasmids

Myc-tagged murine Spred1 cDNA in pcDNA3 and its C-terminal deletion mutants were described previously (12). Murine zDHHC1,5,7,9,12,13,16,20,24 cDNAs were isolated from full-length murine cDNA library of obtained from NIH cloned in pCMV-SPORT6 (35). zDHHC1 and zDHHC24 cDNA were subcloned into pCMV-HA vector for co-IP experiments. tdTomato-labeled p62 and DsRed-labeled Beclin-1 cDNA were generous gifts from Dr Giichi Takaesu, Ryukyu University (36).

### Antibodies

Antibodies used in this study are listed as follows; anti-phospho-ERK (E10 #9106 Cell Signaling), anti-ERK1/2 (137F5 #4695 Cell Signaling), anti-GFP (sc-8334 SANTA CRUZ and NB600–308 Novus Biologicals), anti-FLAG-Tag (M2 Sigma-Aldrich), anti-Tubulin (T9026, clone DM1A Sigma-Aldrich and #2144 Cell Signaling), anti-Flotillin (#18951 IBL Co, Ltd), anti-Calbindin D28K (D-4 Sc-365360 SANTA CRUZ), anti-HA tag (#ab-hatag Invitrogen or Y-11 sc-805 SANTA CRUZ), anti-Spred1 (ab77079 abcam), anti-MAP2 (M4403 Sigma-Aldrich), anti-Myc Tag (sc-789 SANTA CRUZ), Anti-mouse IgG antibody conjugated beads (00–8811–25 TrueBlot Anti-Mouse IgG Agarose Beads (Rockland Immunochemicals), Anti-GFP-agarose (06,083–05 Nacalai Tesque), Anti-Myc-agarose (sc-500771, SANTA CRUZ), Alexa Fluor 546 goat anti-rabbit IgG (H  + L) (A11035 invitrogen), Anti-rabbit IgG, HRP-linked antibody (#7074 Cell Signaling), Anti-mouse IgG, HRP-linked antibody (#7076 Cell Signaling), and Alexa Fluor 647 F(ab’)2 fragment of goat anti-mouse IgG (H  + L) (A21237 Invitrogen).

### Cell culture, transfection and ERK-reporter assay, and luciferase assay

Expression of SPRED1 cDNA in HEK293T cells was described previously (14). The Elk-1 activation was measured by the GAL4 DNA-binding domain (DB)/Elk-1 fusion system according to the manufacturer’s instructions (PathDetect in-vivo signal transduction pathway trans-reporting system, Agilent Technologies) as described (10). In reporter assays, HEK293T cells (7 x 10^4^ cells/well for 24-well plates or 1 × 10^5^ cells/well for 12-well plates) were transfected using PEI MAX (Transfection Grade Linear Polyethylenimine Hydrochloride Polysciences, Inc.) according to the manufacturer’s instruction. HEK293T cells were transfected with 50 ng Elk-1 consisting of GAL4 DB and Elk-1, 50 ng pFR-Luc carrying the GAL4 UAS-fused luciferase gene, 50 ng pCH110 encoding the β-galactosidase gene under the control of the SV40 promoter, and 10 ng FLAG-tagged SPRED1 expression vectors. In some experiments, 10 ng zDHHC expression vectors were included. After 24 h, cells were treated with 50 ng/ml epidermal growth factor (EGF) for 6 h and then collected and lysed with a Reporter lysis buffer (Progmega). The activity of luciferase and β-galactosidase were analyzed using beetle luciferin (Promega) and o-nitrophenyl β-galactopilanoside (Nacalai Tesque) as substrates.

### Western Blotting

Protein from cell lysates was precipitated using 2 μg of anti-FLAG antibody and 25 μl of TrueBlot anti-mouse Ig antibody-conjugated beads or anti-GFP-agarose (Nacalai Tesque), or anti-Myc-agarose (SANTA CRUZ) for 2 h at 4 °C (37). The immune complex was washed three times with a buffer containing 50 mm Tris-HCl (pH 8.0), 150 mm NaCl, and 1% Nonidet P-40. For Western blotting, the immunoprecipitates or whole cell lysates were resolved using SDS-PAGE and transferred to Immobilon-P membranes (Millipore). The membranes were blotted with the indicated antibodies, and the bound antibodies were visualized using horseradish peroxidase-conjugated antibodies against rabbit, or mouse IgG, and Chemi-Lumi One L Western blotting detection reagents (Nacalai Tesque).

### Membrane raft isolation

Membrane raft fraction was obtained as described previously (12). Briefly, Transfected HEK293T cells (5 × 10^6^ cells) were lyzed in 0.3 ml of a Triton X-100 lysis buffer (50 mm Tris-HCl pH 8.0, 10 mm MgCl_2_, 150 mm NaCl, 20 mm NaF, 1 mm Na_3_VO_4_, 1% Triton X-100, 5 mm 2-mercaptoethanol, 5% glycerol, and a protease inhibitor cocktail), incubated on ice for 1 h, and mixed with an equal volume of 80% sucrose in buffer A (50 mm Tris-HCl pH 7.4, 50 mm NaCl, 10 mm MgCl_2_, 1 mm Na_3_VO_4_, and a protease inhibitor cocktail). The mixture was transferred to a centrifuge tube and sequentially overlaid with 0.6 ml of 35% sucrose in buffer A and 0.2 ml of 5% sucrose in buffer A. After centrifugation at 100,000*g* at 4 °C for 16 h, the fractions between the 5% and 35% sucrose interface (raft fraction) were collected.

### Palmitoylation (click chemistry) assay

Metabolic labeling of HEK293T cells with 17-ODYA and subsequent Click chemistry was performed according to the procedure by Butland *et al.* (19, 38). Briefly, 10 h after transfection, 25 μM 17-ODYA (alkyne-containing palmitate analog) (#90270 Cayman Chemical), a palmitate derivative with terminal alkyls complexed with defatted BSA (25%) for an additional 14 h. Then, cells were lysed in PBS containing 1% n-dodecyl β-D-maltoside. Lysate of the cells was immunoprecipitated with anti-GFP antibody-conjugated agarose beads (06,083–05 Nacalai Tesque) to purify EGFP-hSPRED1. Beads were incubated for 90 min at RT in 30 μl of Click chemistry buffer (100 μM tris(benzyltriazolylmethyl) amine, 5 mM CuSO4, 1 mM tris(2-carboxyethyl)phosphine, 500 μM biotin-azide). After the click reaction, samples were eluted from beads by the addition of Laemmli SDS-PAGE buffer, followed by a 30-min incubation at 65 °C. The supernatant was centrifuged and the agarose beads were dropped off, and then the supernatant was subjected to Western Blotting with Streptavidin-HRP (#3999 Cell Signaling) to detect biotin.

### Fluorescence microscopy of transfected cells

Transfected HEK293T cells plated on culture coverslips (354,114 FALCON) were washed in PBS and cells were fixed in 4% Paraformaldehyde Phosphate Buffer Solution (09,154–85 Nacalai) and incubated for 30 min at RT. For 2-BP treatment (21,604 Sigma Sigma-Aldrich), HEK293T cells were incubated with indicated concentrations of 2-BP for 20 h. The stock solution of 2-BP (200 mM) was prepared in 100% Ethanol. The nucleus was stained with Hoechest 33,342 solution (346–07951 DOJINDO). The coverslips were then washed in PBS, air-dried, and subsequently mounted on glass slides, then observed and photographed with a BZ-X700 fluorescence microscope (Keyence).

### Generation of Spred1^P415^ mutant mice by CRISPR/Cas9 system

Mutant mice carrying mSpred1 P415 mutations were generated using the CRISPR-Cas9 system according to a previous report with some modifications (39). For generation of the mutant mice, *pX330* plasmid DNA vector, donor single-stranded oligodeoxynucleotide (ssODN), and fertilized eggs of C57BL/6J mice were used. The sequence (5′-CCATGTATGTGCTGCTACGTCCC -3′) in exon six was selected as the gRNA target, and it was inserted into the entry site of the *pX330*. The donor184 mer ssODN was designed to induce a point mutation of P415A (CCA > GCC) as shown in Fig. S3 (Integrated DNA Technologies). Female C57BL/6J mice were injected with pregnant mare serum gonadotropin and human chorionic gonadotropin at a 48-h interval, and mated with male C57BL/6J mice. We then collected zygotes from oviducts in mated females and a mixture of the *pX330* (circular, 5 ng/μl, each) and the ssODN (10 ng/μl) was microinjected into zygotes. Subsequently, survived injected zygotes were transferred into oviducts in pseudopregnant ICR female and newborns were obtained. Genotypes of the F0 mice were determined by PCR with the following primer sets; *forward* for WT and P415A (5′- AGACATCCGGACATGTGGA -3′), reverse for WT (5′- TGAAATCACTGTCCCTTAGTGC-3′), reverse for P415A (5′- CGTAGCAGCACATACAGGC -3′) to give 939 bp or 475 bp product for WT or P415A, respectively. The candidate F0 mice were mated with the C57BL/6J mice to obtain F1 offspring. Genotypes of F1 mice were determined by PCR, and the PCR products were sequenced. In addition to P415A substitution, P415V substitutions as well as M417Afs∗4 which has 4 bp deletion resulting in C-terminal 28 amino acids (Fig. The F1 mice were obtained. crossed with the C57BL/6J mice to obtain *Spred1*^P415A/+^, *Spred1*^P415V/+^, and *Spred1*^M417Afs∗4/+^ mice.

### Immunohistochemical staining of the brain

Murine brain section and immunohistochemistry with anti-Spred1 antibody (Abcom; ab77079), anti-MAP2 and anti-Calbindin D28K have been described previously (40, 41). Briefly, after perfusion with PBS containing 4%-Paraformaldehyde Phosphate Buffer Solution was injected through the heart. The head of the mouse was then subjected to additional fixation in 4% Paraformaldehyde in PBS at 4 °C overnight. The brain was removed from the mouse, paraffin-embedded as 1 mm wide sagittal or coronal slices, and then sliced to 5 μm using a microtome. Sections were treated with a microwave oven at 750W for 15 min in 10 mM sodium citrate buffer (pH 6.0) to expose antigens and then blocked in Blocking One Histo (NACALAI TESQUE, INC.) at room temperature for 1 h. Primary antibody reaction was performed in 5% BSA in PBS at 4 °C overnight. Samples were treated with secondary antibodies, Alexa Fluor 546-conjugated goat anti-rabbit IgG (H  + L) (Invitrogen, A11035) or Alexa Fluor 647-conjugated F(ab')2 Fragment of goat anti-mouse IgG (Invitrogen, A21237) in PBS containing 5% BSA for 1 h at room temperature. Then slices were mounted in Permafluor (ThermoFisher) with covership and photographed using a BZ-X700 fluorescence microscope (Keyence). Quantification was performed using BZ-H4A software.

### Ataxia phenotype tests

A protocol for the quantification of disease severity in mouse models of cerebella ataxia including hindlimb clasping and footprinting, has been shown as a video (42). Mice were placed on a corridor and allowed to move freely across the walkway. Three uninterrupted runs were obtained for each mouse. Then, the distances between the two forepaws were then measured perpendicular to the direction of walking.

### Sex as a biological variable

Our study examined both male and female animals, and similar results are reported for both sexes. However, for behavioral testing, we used males only to avoid sex bias.

### Spermidine (SPD) treatment

SPD (3 mM final concentration) was prepared from aqueous stock solutions (0.6 M, adjusted to pH of 7.0–7.5), and was administered *ad libitum via* autoclaved drinking water. Control animals received regular autoclaved drinking water. SPD-supplemented drinking water was freshly prepared every 3 to 4 days. For preparation of SPD stock solutions “spermidine free base” (Sigma-Aldrich #85558) was carefully titrated (slowly on ice to prevent the solution from heating up) to pH 7.0 to 7.5 using hydrochloric acid. The pH-adjusted stock solution was then sterile filtered and stored for up to 3 months at −80 °C in single-use aliquots (25, 27).

## Data availability

All data and methods used to support the conclusions of this study are available in the main text, figures, and supplementary information. Any additional data related to this work are available from the corresponding author, Akihiko Yoshimura (yoshimura@keio.jp) upon request.

## Supporting information

This article contains supporting information.

## Conflict of interest

The authors declare that they have no conflicts of interest with the contents of this article.
