# Supplementary material for: Legius syndrome mutations in the Ras-regulator SPRED1 abolish its membrane localization and potentially cause neurodegeneration
Source: J Biol Chem. 2024 Nov 5;300(12):107969. doi: 10.1016/j.jbc.2024.107969 (PMC11648228; doi:10.1016/j.jbc.2024.107969)
Supplement: Supporting information [file mmc1.pdf]

Summary of Pathogenesis of mutations

https://grenada.lumc.nl/LOVD2/mendelian_genes/variants.php?select_db=SPRED1&action=search_all&order=Variant/RNA,ASC&hide_col=&show_col=all

I234M	p.(Ile234Met)	KBD	unknown	dbSNP: rs138553244	unknown	nearly 12-yo boy with 1-5 irregular CALs, L axillary freckling, family history unknown, African American
I234M	p.(Ile234Met)	KBD	de novo	Messiaen UAB, unpublished	unknown	2.5-yo boy with >6 typical CALMs over anterior aspect and a large hyperpigmented patch over L buttock, spor.
R256H	p.Arg256His	KBD	unknown	Brems et al, 2012	unknown	-
R257C	p.Arg257Cys	KBD	unknown	Brems et al, 2012	Legius Syndrome	4-year 3 CALMs
Y258C	p.Tyr258Cys	KBD	unknown	Messiaen UAB, unpublished	Legius Syndrome	3-y with 13 typical CALMs, bilateral inguinal freckling
Y258C	p.Tyr258Cys	KBD	unknown	Messiaen UAB, unpublished	Legius Syndrome	8-yo, >10 CALMs, inguinal freckling, macrocephaly, learning disabilities, bilateral Unidentified bright objects
Y261D	p.(Tyr261Asp)	KBD	de novo	Messiaen UAB, unpublished	Legius Syndrome	no signs in both parents
V309A	p.Val309Ala	-	de novo	Spencer 2011	unknown	3.5-y >6 CALMs, bilateral axillary freckling, pulmonic stenosis
V309A	p.Val309Ala	-	familial	Spencer 2011	unknown	8.5-yo boy with 3 typical CALMs and ADD, familial
V309A	p.Val309Ala	-	unknown	Spencer 2011	Neurofibromatosis type 1	7-mo boy with multiple CALMs, FH? Carries a pathogenic truncating NF1 mutation
P311L	p.Phe311Leu	-	familial	Messiaen UAB, unpublished novel mutation	multiple CALMs	5-yo girl with 4 typical CALMs predominantly on R arm, segmental NF is possible, spor. Same missense mutation was found in the unaffected father
R334C	p.Arg334Cys	SPR	familial	Messiaen UAB, unpublished novel mutation	1 CALM	5.5-yo, 1 CALM, MRI T2-weighted hyperintense globus pallidus left; mother carries same SPRED1 mutation and no signs
H344R	p.His344Arg	SPR	unknown	Spencer 2011	unknown	-
D398N	p.(Asp398Asn)	SPR	unknown	Messiaen 2009	unknown	1-year old, >6 CALMs, unknown family history
V408E	p.Val408Glu	SPR	unknown	Brems et al, 2012	unknown	-
P415A	p.(Pro415Ala)	SPR	familial?	Messiaen 2009	Legius Syndrome	4-y, 12 CALM, 7 mm diameter CAL in axilla, language delay, fine motor delay
P415A	p.(Pro415Ala)	SPR	familial	Messiaen 2009	Legius Syndrome	12-y, >6 CALM, no freckling
C416R	p.Cys416Arg	SPR	unknown	Spencer 2011	unknown	5-year, >6 CALMs, left inguinal freckling; mother also has CALMs and freckling
C416R	p.Cys416Arg	SPR	unknown	Spencer 2011	unknown	17-yo boy with >6 CALMs, R inguinal freckling, FH?? Mother was tested and does not carry C416R although she has 1-5 typical CALs, father not yet tested
C418R	p.Cys418Arg	SPR	familial	Messiaen UAB, unpublished	Legius Syndrome	4-mo, >6 typical CALMs, right axillary freckling, abnormal development; mother and maternal grandmom also reportedly have CALMs
M425V	p.Met425Val	SPR	unknown	Brems et al, 2012	Legius Syndrome	10-year >6 CALMs, bilateral inguinal, atypical right axillary freckling
G430A	p.Gly430Ala	SPR	familial	Brems et al, 2012	Neurofibromatosis type 1	this SPRED1 mutation was found in a father and son who both also carried a clearcut pathogenic and recurrent NF1 mutation
C433Y	p.(Cys433Tyr)	SPR	unknown	Messiaen 2009	unknown	-
A442T	p.Ala442Thr	SPR	familial	Messiaen UAB, unpublished	familial CALM and freckling	7-yo boy with >6 CALMs and skin fold freckling. Fam. Also carries NF1 c.4769G>T; p.R1590Leu - mom is 38yo with multiple CALMs and skin fold freckling and also carries BOTH the NF1 and SPRED1 variant

Supplementary Figure 1

Phenotypic information on patients with missense mutations in the KBD and SPR domains of SPRED1. Information was transferred from https://grenada.lumc.nl/LOVD2/mendelian_genes/variants.php?select_db=SPRED1&action=search_all&order=Variant/RNA,ASC&hide_col=&show_col=all

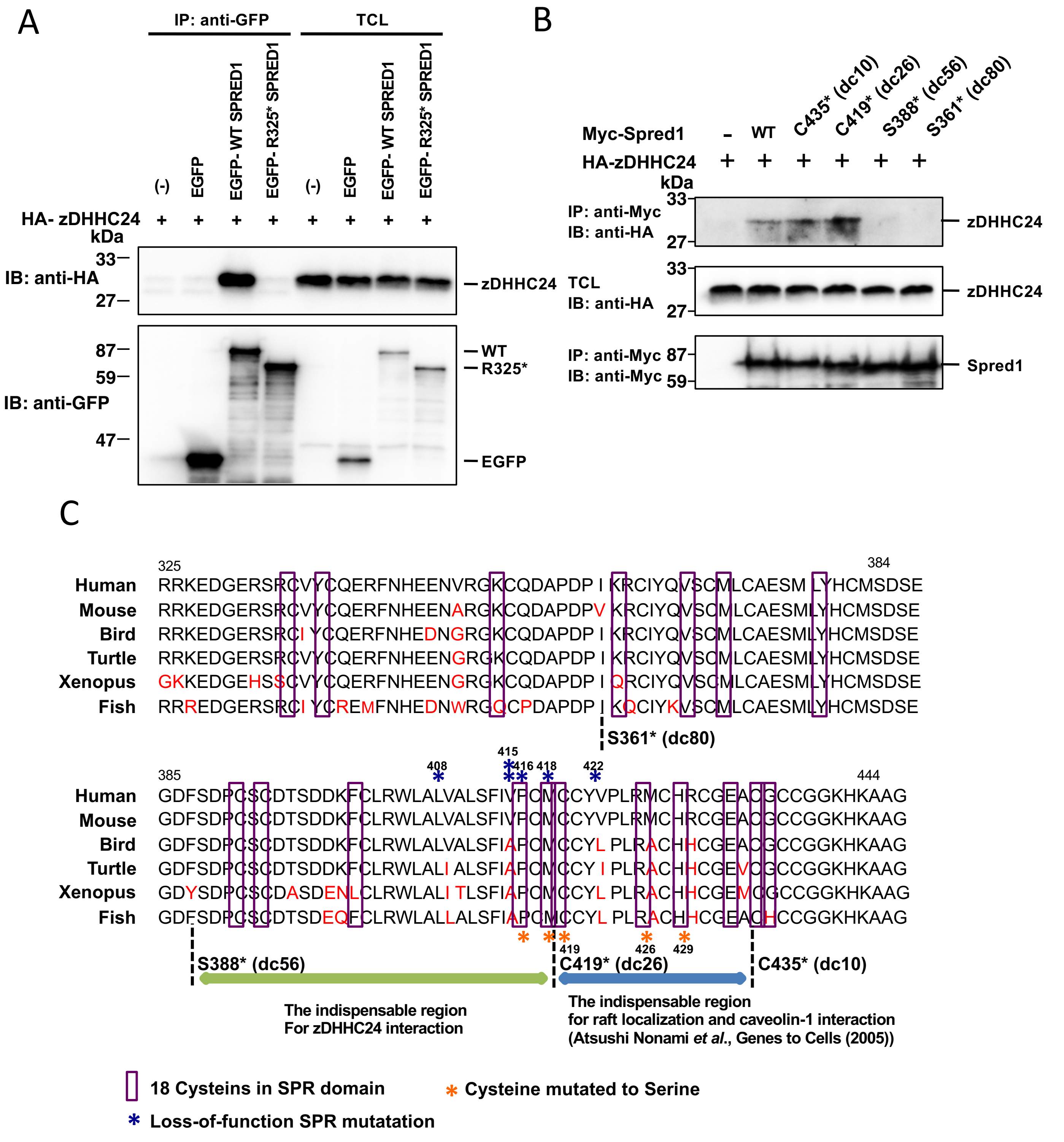

Supplementary Figure 2

Binding of HA-zDHHC24 to EGFP-hSPRED1 (A) and murine Myc-tagged murine Spred1 C-terminal deletion mutants (C435*, C419*, S388*, S361*). (B). TCL; total cell lysate. HEK293T cells were transfected with the indicated plasmids and immunoprecipitated with anti-GFP (A) or anti-Myc antibodies (B), followed by immunoblotting with indicated antibodies. (C) Amino acid sequence comparison of the SPR domain of SPRED1 from different species. Positions of C-terminal deletion mutants described in ref.12 are shown. Sequences are from Human (AAP59414.1), Mouse (NP_277059.1), Bird (Gallus galus, NP_001186638.1), Turtle (Chelonia mydas, XP_007069456.1), Xenopus (Xenopus tropicalis, AAU43767.1), Fish (Aphyosemion striatum, SBP09301.1).

Spred1P415A CRISPR-Cas9

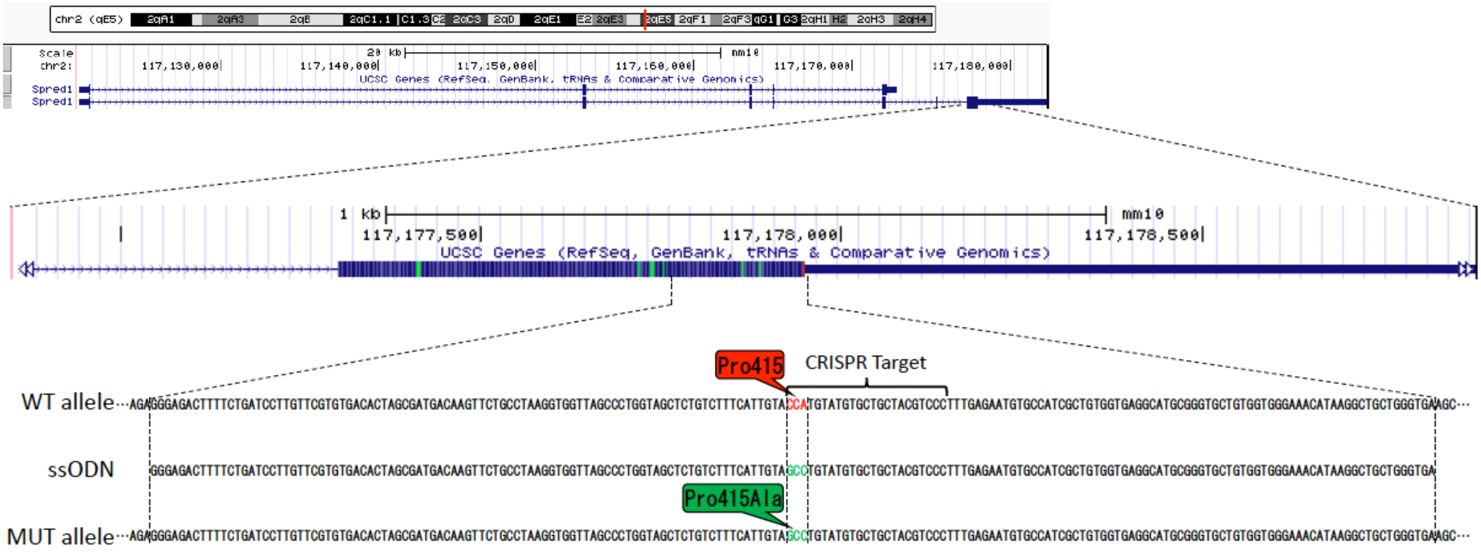

Supplementary Figure 3

The structure of the murine Spred1 gene and the nucleic acid sequences of single-stranded oligodeoxynucleotide (ssODN) for the knock-in. The predicted mutant P415A substitution is shown at the bottom.

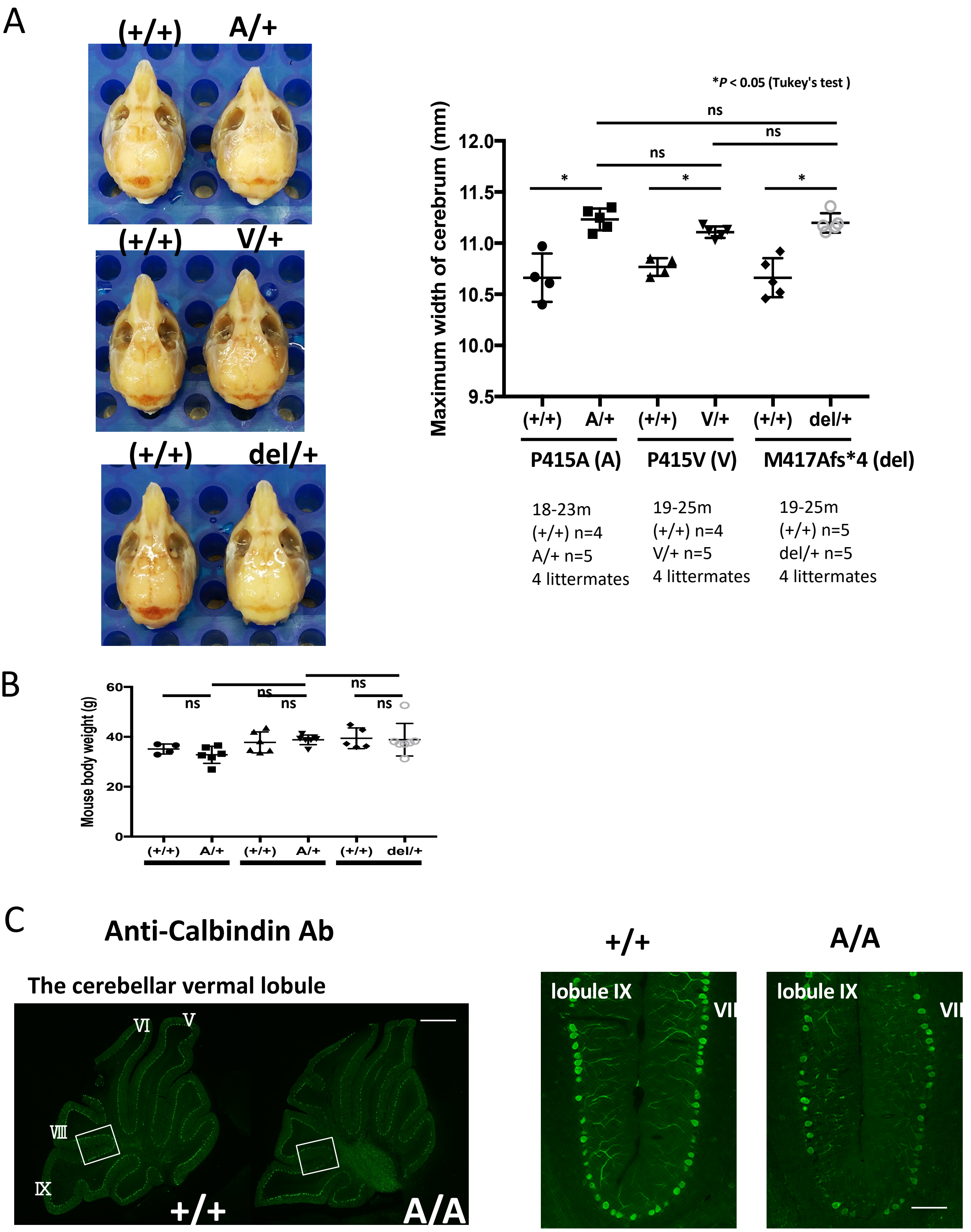

Supplementary Figure 4

Characterization of KI mice. (A) Skull appearance and maximum width of the cerebrum in heterozygous mutant mice and their littermates (+/+). Brain size was measured in male littermates of both sexes at around 19 months of age. Measurements were made on brains fixed in 4% paraformaldehyde-PBS buffer. The head was then post-fixed at 4° C for 18 hours before the brain was removed. The size of the cerebrum of the removed brain was measured using calipers (DIGITAL CALIPER DT-150 Niigataseiki). (B) Body weight of 17-21month old mice (shown in **Fig.7B**). (C) Detection of Purkinje cells in the cerebellum by immunohistochemistry with anti-calbindin D28K antibody. The brain was cut sagittally for paraffin sectioning. The number of cerebellar vermis lobules is expressed as a Roman number. Bars; 500 µm (left panel) and 100 µm (right two panels), respectively.

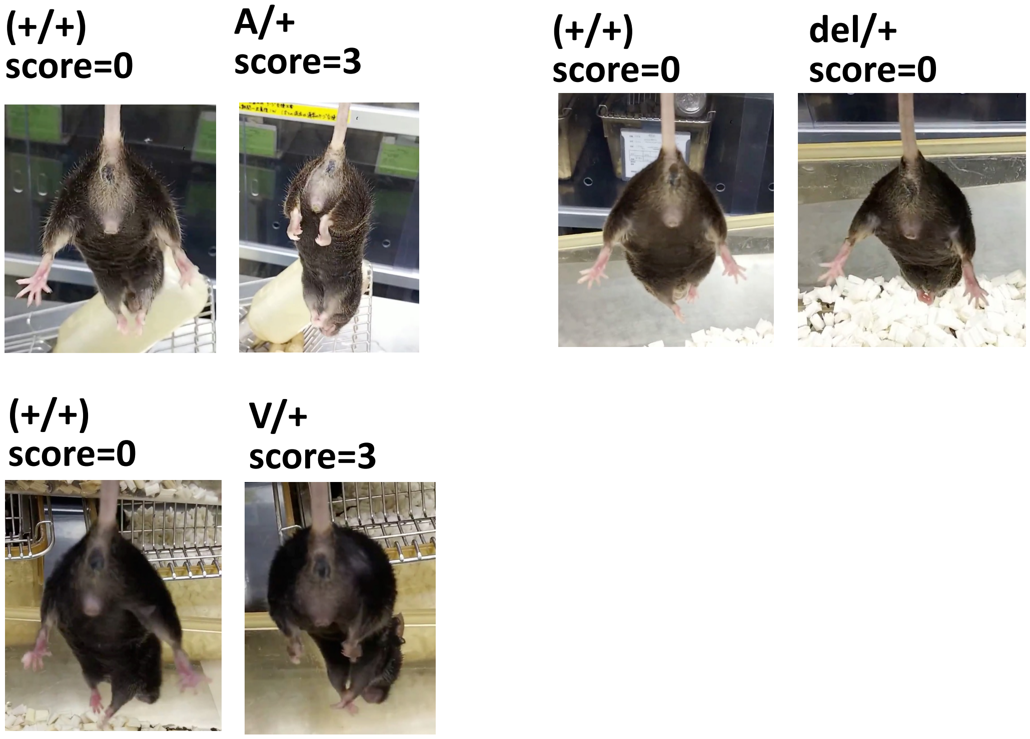

Supplementary Figure 5

Representative image of the hind limb grasping test with score 0 or 3. Hindlimb clasping score was determined by grasping and lifting the tail of the mouse (14 months old) and observing the condition of the hind legs for at least 10 s, and was measured at least three times on different days within the same time window of 15:00-18:00 h in all mice. The numbers of mice we examined are shown in **Fig.7A**.

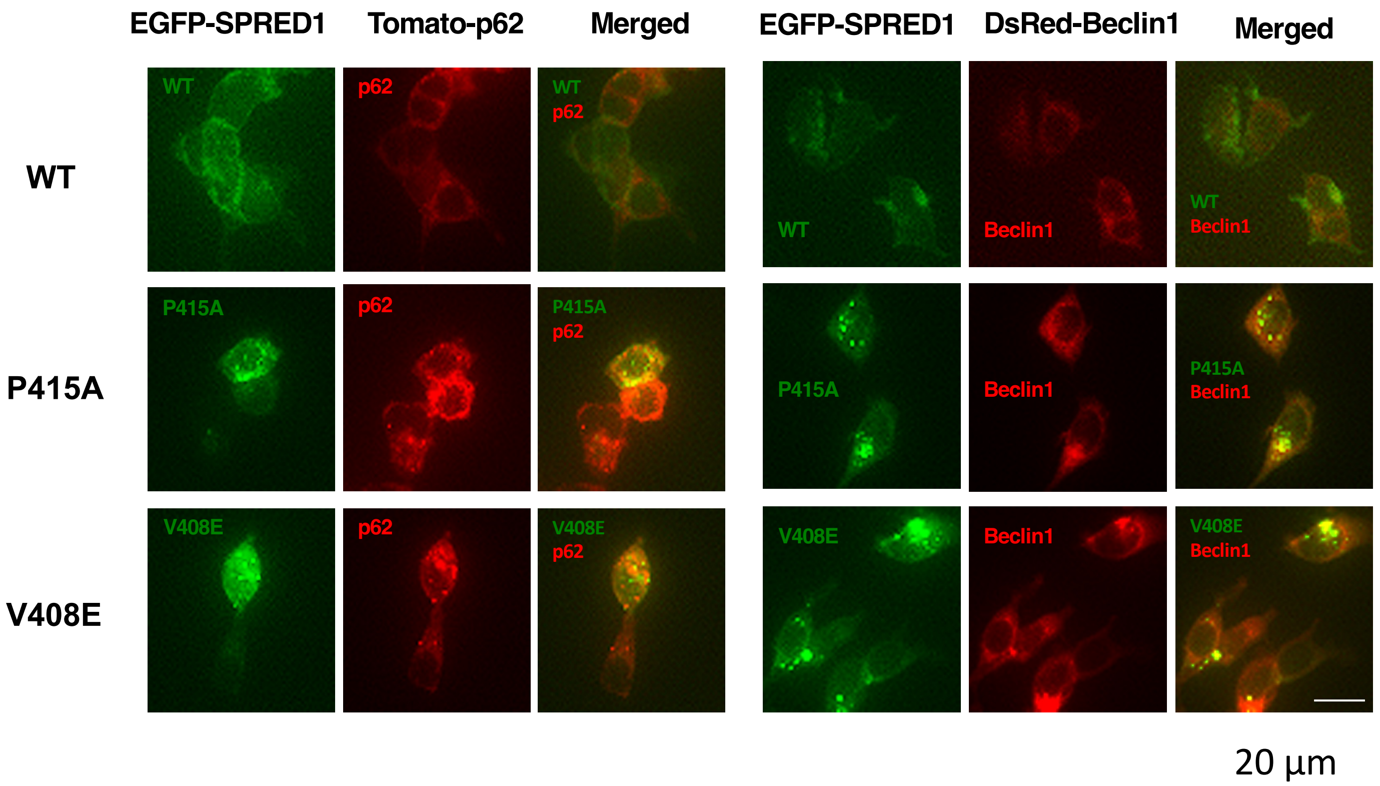

Supplementary Figure 6

Co-localization of P415V and V408E EGFP-SPRED1 with tdTomato-labeled p62 and DsRed-labeled Beclin-1. The indicated plasmids were transfected into HEK293 cells and examined by fluorescence microscopy 24h after transfection.

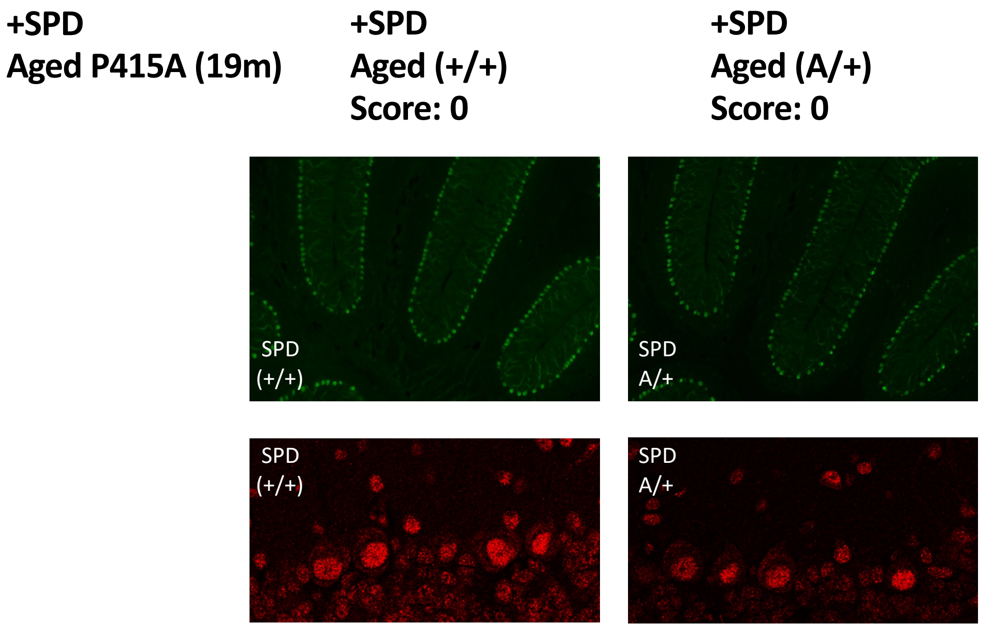

Supplementary Figure 7

Effect of SPD on Purkinje cells of 19-month-old (+/+) or (A/+) mice detected by immunohistochemistry with anti-calbindin D28K antibody (Green). Spred1 protein was detected with anti-Spred1 antibody (Red). Quantitative data are shown in **Fig.8B**.
